# Toxigenic *Corynebacterium ulcerans* in Woman and Cat

**DOI:** 10.3201/eid1709.110391

**Published:** 2011-09

**Authors:** Anja Berger, Ingrid Huber, Sophie-Susann Merbecks, Ingrid Ehrhard, Regina Konrad, Stefan Hörmansdorfer, Michael Hogardt, Andreas Sing

**Affiliations:** Author affiliations: National Consiliary Laboratory for Diphtheria, Oberschleißheim, Germany (A. Berger, R. Konrad, A. Sing);; Bavarian Health and Food Safety Authority, Oberschleißheim (A. Berger, I. Huber, R. Konrad, S. Hörmansdorfer, M. Hogardt, A. Sing);; Public Health Laboratory of Saxony, Germany (S.-S. Merbecks, I. Ehrhard)

**Keywords:** diphtheria, zoonosis, diphtheria toxin, human, cat, bacteria, letter

**To the Editor:** Diphtheria and diphtheria-like illness are caused by *Corynebacterium* spp. that harbor the diphtheria toxin–encoding *tox* gene. Recently in many industrialized countries, cases of diphtheria-like infection caused by toxigenic *C. ulcerans* have outnumbered those caused by toxigenic *C. diphtheriae* (*1**,**2*). *C. ulcerans* infection was originally associated with consumption of raw milk and dairy products or contact with cattle, but *C. ulcerans* has increasingly been isolated from domestic animals such as pet dogs and cats (*3**–**5*). So far, isolation of an identical toxigenic *C. ulcerans* strain from an animal and its owner has been documented only for dogs (*3**,**4*) and a pig (*6*). We report the isolation of an identical toxigenic *C. ulcerans* strain from an asymptomtic pet cat and a person with pharyngeal diphtheria-like illness; therefore, it might be speculated that the woman has acquired her infection from the cat.

In November 2010, an 86-year-old woman with arterial hypertension and rheumatoid arthritis was admitted to an ear, nose, and throat clinic in Dresden, Germany, with a 3-day history of sore throat, hoarseness, and nasal respiratory obstruction. Fever was not reported. Because the patient had visible fibrinous rhinitis, a nasal and pharyngeal swab was obtained before treatment with amoxicillin was begun. The patient had no history of recent travel abroad or contact with livestock. Her complete vaccination status against diphtheria was unknown, but she had received a vaccination booster in 2006.

Toxigenic *C. ulcerans* grew from culture of the nasal swab specimen; it was identified by biochemical differentiation (API Coryne code 0111326; bioMèrieux, Nürtingen, Germany), *rpoB* sequencing (*6*), and MALDI-TOF analysis (MALDI Biotyper; Bruker Daltonics, Bremen, Germany) (*7*). Toxigenicity was verified by real-time PCR (*8*) and a modified Elek test (*6*).

Because the microbiological result suggested diphtheria-like illness, the patient was transferred to an infectious diseases department in an academic hospital, where she was isolated and treated with amoxicillin for 12 days. Because the patient’s condition was stable and no severe complications occurred during her hospital stay, she was not given diphtheria antitoxin. Her predominant symptoms, such as sore throat and earache, improved after antimicrobial drug therapy, and she recovered quickly. Electrocardiogram performed before discharge from hospital showed no signs of myocarditis or other toxin-related effects, such as neurologic disorder.

Although person-to-person transmission of *C. ulcerans* has not yet convincingly been demonstrated, an outbreak investigation involving the patient’s close contacts (6 family members, the physician, and 19 nurses and other health care workers) was conducted. Although all close contacts had completed the series of diphtheria toxoid vaccinations, they were all given postexposure prophylaxis with erythromycin.

Because of the zoonotic potential of human *C. ulcerans* infections, nasal and pharyngeal swab samples were collected from the patient’s asymptomatic pet cat. Strains of *tox*-positive *C. ulcerans* (which we named KL251 and KL252) grew on culture; the API Coryne code was identical to that of the human isolate KL246. In contrast to the human isolate, which yielded a weakly positive Elek result, both isolates from the cat showed Elek-negative results.

Antimicrobial drug susceptibility testing of the 3 isolates was performed on Mueller-Hinton blood agar (supplemented with 5% sheep blood) by using the Etest system after overnight incubation at 37°C and in 5% CO_2_. In the absence of standardized breakpoints for *C. ulcerans*, susceptibility was determined by using the Clinical Laboratory Standards Institute criteria for broth microbouillon dilution susceptibility testing for *Corynebacterium* spp (*9*). All *C. ulcerans* strains were susceptible to amoxicillin, benzyl penicillin, ceftriaxone, erythromycin, and tetracycline (MICs 0.19–0.5 µg/mL) but less susceptible to clindamycin in vitro (MIC 2 µg/mL).

Sequencing of *rpoB* and *tox* showed 100% homology between the strains from the woman and the cat. Ribotyping revealing a U3-like ribotype (*5*), and multilocus sequence typing (*10*) confirmed the clonal identity of the strains.

The cat was given a combined preparation of benzyl penicillin and streptomycin. After completion of therapy, *C. ulcerans* no longer grew from nasal swab specimens from the woman or the cat*.*

Our findings of transmission of toxigenic *C. ulcerans* between a woman and her cat underline the zoonotic potential of this organism and highlight the need for more studies investigating the carrier status of companion animals such as cats and dogs. Although clindamycin is not a first-line drug for diphtheria therapy, the intermediate susceptibility of *C. ulcerans* against clindamycin underscores the necessity of standardized susceptibility testing for diphtheria cases because clindamycin-resistant toxigenic *C. ulcerans* strains in human infections have been recently reported (*6*). Toxigenic *C. ulcerans* strains are rare, but the numbers of human wound infections or diphtheria-like disease caused by *C. ulcerans* have increased in the past few years. However, detection of toxigenic *C. ulcerans* is often still incidental, often resulting in delayed specific therapy, including patient isolation or contact tracing.
